# SARS-CoV-2 Omicron variant: recent progress and future perspectives

**DOI:** 10.1038/s41392-022-00997-x

**Published:** 2022-04-28

**Authors:** Yao Fan, Xiang Li, Lei Zhang, Shu Wan, Long Zhang, Fangfang Zhou

**Affiliations:** 1 School of Medicine, Zhejiang University City College, 310015 Hangzhou, Zhejiang China; 2 Institutes of Biology and Medical Science, Soochow University, 215123 Suzhou, PR China; 3 The Eighth Affiliated Hospital, Sun Yat-sen University, 518033 Shenzhen, China; 4 MOE Laboratory of Biosystems Homeostasis & Protection and Innovation Center for Cell Signaling Network, Life Sciences Institute, Zhejiang University, 310058 Hangzhou, China; 5 Department of Orthopaedic Surgery, The First Affiliated Hospital of Wenzhou Medical University, 325000 Wenzhou, China; 6 Brain Center, Affiliated Zhejiang Hospital, Zhejiang University School of Medicine, 310058 Hangzhou, China

**Keywords:** Infectious diseases, Infectious diseases

## Abstract

Since the outbreak of the coronavirus disease 2019 (COVID-19) pandemic, there have been a few variants of the severe acute respiratory syndrome coronavirus 2 (SARS-CoV-2), one of which is the Omicron variant (B.1.1.529). The Omicron variant is the most mutated SARS-CoV-2 variant, and its high transmissibility and immune evasion ability have raised global concerns. Owing to its enhanced transmissibility, Omicron has rapidly replaced Delta as the dominant variant in several regions. However, recent studies have shown that the Omicron variant exhibits reduced pathogenicity due to altered cell tropism. In addition, Omicron exhibits significant resistance to the neutralizing activity of vaccines, convalescent serum, and most antibody therapies. In the present review, recent advances in the molecular and clinical characteristics of the infectivity, pathogenicity, and immune evasion of Omicron variant was summarized, and potential therapeutic applications in response to Omicron infection were discussed. Furthermore, we highlighted potential response to future waves and strategies to end the pandemic.

## Introduction

**Fig. 1:**
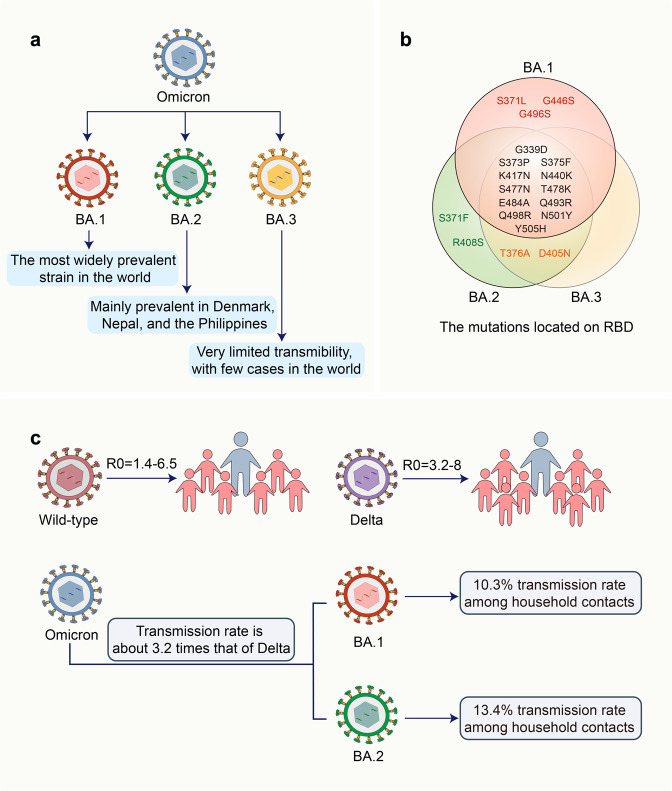
The sub-lineages of the SARS-CoV-2 Omicron variant. **a** The Omicron variant has evolved into three sub-lineages: BA.1, BA.2, and BA.3. **b** Venn diagram showing the mutations located on the S protein RBD of BA.1, BA.2, and BA.3. **c** Transmission speed of SARS-CoV-2 wild-type, Delta, and Omicron variants

The Omicron variant of severe acute respiratory syndrome coronavirus 2 (SARS-CoV-2) was first identified in South Africa and Botswana and was reported to the World Health Organization (WHO) on November 24, 2021, as a novel variant. This novel variant, also known as B.1.1.529, spreads rapidly and was classified as a variant of concern (VOC) by the WHO on November 26, 2021.^1,2^ Further examinations suggest that the Omicron variant did not develop from one of the earlier known variants, as evidenced by several differences between their genomes. Three possible explanations have been proposed for the development of the Omicron variant: silent evolution in a population with little sequencing, long-term evolution in one or a few persons with chronic infection, or evolution in other animals especially rodents.^3^ Notably, the Omicron variant is not a single strain, but evolved into three lineages: BA.1, BA.2, and BA.3. BA.1 was once the most widely prevalent strain in the world; however, BA.2 is gradually replacing BA.1 in several countries, such as Denmark, Nepal, and the Philippines. The transmissibility of BA.3 is very limited, with very few cases, at most a few hundred cases (Fig. 1a).

The Omicron variant has caused global panic and concern owing to its contagious and vaccine-escape mutations. Presently, up to 60 mutations have been identified in the BA.1 lineage, with as many as 38 of these occurring in the spike (S) protein, one in the envelope (E) protein, two in the membrane (M) protein, and six in the nucleocapsid (N) protein (https://covid19dashboard.regeneron.com). BA.2 lineage possesses 57 mutations, with 31 in the S protein, of which the N-terminus is significantly different from that of BA.1. The receptor-binding domain (RBD) of the S protein is responsible for binding to the host receptor angiotensin-converting enzyme 2 (ACE2) and has the potential to increase infectivity and mediate escape from vaccine-induced neutralizing antibodies.^4–6^ Therefore, mutations located in the RBD of the S protein have attracted significant research attention. BA.1 and BA.2 share 12 mutations in the RBD, including G339D, S373P, S375F, K417N, N440K, S477N, T478K, E484A, Q493R, Q498R, N501Y, and Y505H. S371L, G446S, and G496S were only identified in BA.1, whereas R346K was found in a member of this group, namely BA.1.1. BA.2 possesses two unique mutations in RBD, including S371F and R408S, and shares T376A and D405N with BA.3 (Fig. 1b). Some of these mutations have also been found in previous variants and are known to lead to increased transmissibility, higher viral binding affinity, and antibody escape.^7,8^ For instance, mutations in the residues K417, E484, and N501, which have also been found in Beta (B.1.351) and Gamma (P.1) variants, have been suggested to mediate escape from vaccine-induced neutralization.^5^ The effects of most of the remaining Omicron mutations are unknown; thus, our understanding of the viral behavior and susceptibility of the Omicron variant to natural and vaccine-mediated immunity remains unclear. Moreover, individuals previously infected with other SARS-CoV-2 variants can be reinfected with this new variant.^9^ A recent study suggested that the Omicron variant is likely to infect individuals recovering from infections by previously prevalent variants.^10,11^ This evidence shows that mutations in Omicron evade immunity induced by the previous infection.

Given that the Omicron variant poses a serious threat to public health and could undermine global efforts to control the COVID-19 pandemic, there is an urgent need for in-depth studies and a comprehensive understanding of Omicron. Recently, several achievements have been made in understanding the Omicron variant. In this review, we summarized the recent progress in research on the characteristics of the Omicron variant, including its enhanced infectivity and transmissibility, reduced pathogenicity, and immune evasion ability. In addition, we discussed the effectiveness of existing vaccines, neutralizing antibodies, and antiviral drugs and highlighted possible response strategies to Omicron and future variants.

## Infectivity and transmissibility

SARS-CoV-2 utilizes the S protein to bind to the main receptor ACE2 on the host-cell surface and enters the host cell through membrane fusion with the help of furin and type II transmembrane serine protease (TMPRSS2) or cathepsin L,^12^ which is a crucial process of infection. The Omicron variant shares a similar process of infection but is more contagious than previous variants. The findings of preliminary studies on the infectivity and transmissibility of Omicron are discussed below.

### Binding to host receptor ACE2

ACE2 is a major receptor of SARS-CoV-2.^13^ The binding affinity of ACE2 for the S proteins of Omicron is one of the main factors determining viral infectivity. To date, the results on the binding affinity of the Omicron variant to ACE2 are slightly different, possibly owing to differences in experimental materials or methods. Moreover, the aggregation state of the protein has a significant impact on measurement results.^14^ Several studies have shown that the binding affinity of Omicron RBD to ACE2 is approximately 1.5–2.8 times that of the wild-type (WT).^11,15–17^ In contrast, some studies have suggested that the binding ability of Omicron RBD to ACE2 is comparable to that of WT.^18,19^ Compared with the previously prevalent Delta variant, Omicron RBD exhibits a similar or weaker binding ability to ACE2.^11,17–19^ In addition, the binding ability of Omicron RBD to ACE2 is much weaker than that of Alpha variant, with only one mutation, N501Y, in the RBD.^16,19^ Based on the above studies, it can be inferred that the binding ability of Omicron RBD to ACE2 is roughly between that of WT and Delta RBD. S477N, T478K, Q493R, Q496S, and Q498R have been reported to potentiate the interaction between Omicron and ACE2 by establishing new hydrogen bonds or salt bridges with the corresponding sites of ACE2^14,15,17,19^ in addition to N501Y. However, K417N and E484A can cause a significant loss of polar interactions between Omicron and ACE2, offsetting some of the enhanced interactions forged by other mutations.^14,15,17,19^

Overall, mutations in the Omicron RBD did not affect its receptor recognition and binding to ACE2, and the Omicron RBD can efficiently bind to human ACE2 for host-cell entry. Notably, the Omicron S protein can bind to human ACE2 or ACE2 orthologs of different animal species to enter the target cells.^20^ These findings indicate the zoonotic potential of the Omicron variant, which could contribute to the development of highly infectious variants.

### Host-cell entry

The entry of SARS-CoV-2 into the host cells after binding to host receptors is mediated by the S protein. The S protein is composed of S1 and S2 subunits.^21^ The S1 subunit contains the RBD, which binds to ACE2, whereas the S2 subunit contains the transmembrane portion of the S protein, which is responsible for anchoring the S protein to the membrane and mediating fusion of the viral membrane with cellular membranes.^22,23^ Cleavage of the S protein at the S1–S2 and S2 sites, mediated by furin^24^ and type II transmembrane serine protease (TMPRSS2)^25^or cathepsin L,^26^ is crucial for viral entry into host cells. Cleavage by TMPRSS2 and cathepsin L at the S2 site mediates two distinct SARS-CoV-2 entry pathways. As TMPRSS2 is present on the cell surface, TMPRSS2 mediates the plasma membrane route of entry, whereas cathepsin L in the endosome mediates the endosomal entry route.^22^

**Fig. 2:**
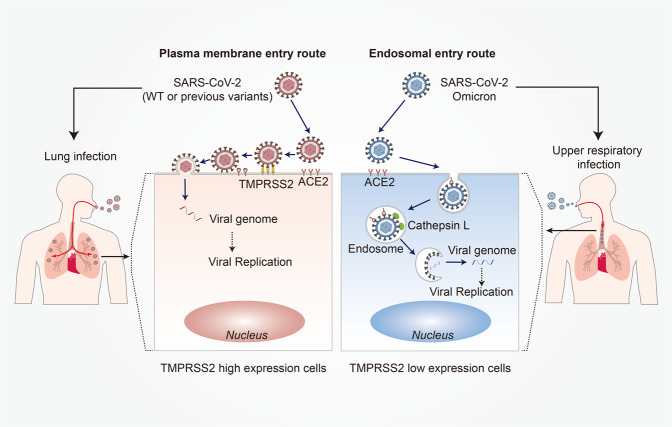
The different entry routes and pathogenesis between SASR-CoV-2 WT or previous variants and Omicron variant. Left: SASR-CoV-2 WT or previous variants mainly infect lung epithelial cells, which are TMPRSS2 high expressed cells, and enter host cells by plasma membrane route. In the plasma membrane entry route, virus first binds to ACE2, then binds to TMPRSS2 and is cleaved at the S proteins. Next, the S protein anchors to the cell membrane and mediates fusion of the viral membrane with the cell membrane. Finally, a pore is formed in the membrane and the viral genome is released into the cell. Right: SASR-CoV-2 Omicron variant mainly infects the upper airway epithelial cells, which are TMPRSS2 low-expressed cells, and enter host cells by the endosomal route. In the endosomal entry route, a virus first binds to ACE2 and the virus–ACE2 complex is internalized via endocytosis into the endosomes, where S protein is cleaved by Cathepsin L. Then the viral and endosomal membranes are fused together to form a pore and release the viral genome

The Omicron variant harbors six unique mutations on S2 (N764K, D796Y, N856K, Q954H, N969K, and L981F) that have not been identified in previous VOC.^27^ Recent studies have reported that Omicron spike pseudotyped virus infection was reduced in TMPRSS2 expressing cells, but increased in cells that support endosomal entry, and that the Omicron variant prefers the endosomal entry route rather than the plasma membrane entry route.^28–30^ These findings suggest that mutations on the Omicron S protein non-RBD may alter the route of viral entry into host cells, which is associated with a shift in cellular tropism away from TMPRSS2 expressing cells, and explains the faster replication of Omicron in the upper airways than in the lungs, unlike that of other variants^27,30–32^ (Fig. 2). In addition, the Omicron variant contains three mutations in the furin cleavage site region (P681H, H655Y, and N679K). The basic mutation P681H in the polybasic cleavage site (PBCS), also present in Alpha and Gamma, has been demonstrated to promote furin-mediated cleavage of the S protein, potentially enhancing infectivity.^33^ However, the cleavage level of Omicron by furin is the weakest among the SARS-CoV-2 variants, indicating that other mutations near the furin cleavage site may severely interfere with its cleavage.^34^ Moreover, the fusion ability of the Omicron variant is the slowest among all SARS-CoV-2 variants,^15,27,32^ similar to SARS-CoV-1.^34^

### Transmissibility

The high transmissibility of the Omicron variant is a major cause of global concern. Since the advent of Omicron, it has rapidly replaced Delta as the dominant strain worldwide. In the US, Delta accounted for 99% of new cases on December 4, 2021; however, Omicron accounted for more than 95% by January 8, 2022.^35^ The basic reproduction number (R0) of the Delta variant was between 3.2 and 8.^36^ The transmissibility of the Omicron variant is ~3.2 times that of Delta, and the doubling time is approximately three days.^37,38^ Generally, BA.2 is ~1.4 times more transmissible than BA.1,^39,40^ with a transmission rate of ~13.4% among household contacts, whereas that of BA.1 was 10.3%^41^ (Fig. 1c).

The rapid spread of the Omicron variant is mainly due to its immune evasion ability, which is responsible for the infection of vaccinated and previously infected individuals.^42^ In addition, changes in cell entry and cellular tropism in the Omicron variant may also facilitate rapid transmission.^27,28,31,43^ Moreover, the Omicron variant has been shown to cause more asymptomatic infections than the other variants, which may contribute to the silent spread of the virus.^44^ Furthermore, the binding affinity of Omicron RBD to ACE2 contributes to transmission, but is not a major factor. However, whether Omicron infection can lead to higher viral loads remains controversial.^45,46^

## Pathogenicity

The disease severity of the Omicron variant has sparked extensive discussion and has profoundly affected public policies. Growing evidence has shown that Omicron-infected patients exhibit milder symptoms than those infected by the earlier variants. Moreover, the Omicron variant is more likely to infect the upper respiratory tract and less able to cause lung infection. However, the observed reduction in pathogenicity is now amplified by enhanced immunity. Research from the UK shows that three doses of vaccination caused over 50% reduction in the odds of hospitalization with Omicron compared to those who were not immunized.^47^ Previous infection can also provide similar protection.^48^ The proportion of breakthrough infections caused by Omicron is much higher than that of previous variants, which would reduce the severity we observed. Therefore, the Omicron is still risky for unvaccinated individuals, especially the aged. The treatment of Omicron should be taken seriously.

### Clinical symptoms

Analysis of the ZOE COVID study showed that the most common symptoms of Omicron infection were runny nose, headache, fatigue (mild or severe), sneezing, and sore throat.^49,50^ Generally, there are few differences between the symptom profiles of Omicron and Delta, with a lower occurrence of the classic symptoms of fever, cough, or loss of sense of smell or taste in Omicron-infected patients.^49,50^ There have been few cases of convulsions in children, but the cases were too few to conclude that they were the consequences of the infection.^51^ Furthermore, laboratory studies showed that the mutations in the Omicron variant altered the tropism of the virus. The Omicron variant exhibited a lower replication rate in lung and gut cell lines,^27^ but replicated faster in primary cultures of human nasal epithelial cells.^28^ Consistently, Omicron has been shown to replicate rapidly in human airway organoids and ex vivo explant cultures of human bronchus, but less efficiently in human alveoli organoids and ex vivo explant cultures of the human lung.^31,43^ These results revealed that Omicron tended to infect the upper respiratory tract, but not the lungs, which may contribute to enhanced transmissibility and better prognosis. Most studies attributed this to the inefficient TMPRSS2 usage of the Omicron variant.^27,28,31,43^ However, enhanced infection in the upper respiratory tract was not proven in rodent models, indicating that this point needed more evidence.^32,52,53^

### Severity

A few real-world studies have indicated that the Omicron variant may be milder than earlier variants. A study of early cases in the Gauteng province of South Africa showed that the hospitalization rate during the fourth wave (Omicron-dominated) was ~4.9%, which was markedly below the rate in waves dominated by Beta or Delta variant, and the probability of severe illness was reduced by 73% in the Omicron-dominated wave.^54^ Outcomes in the Western Cape province of South Africa were similar, and the risk of severe hospitalization or death was reduced by ~25%.^55,56^ Moreover, patients infected by the Omicron variant and identified by the S gene target failure (SGTF) had significantly lower odds of hospitalization and severe disease than those by Delta.^57^ Several analyses of patients in the UK showed that the risk of hospitalization with Omicron was approximately one-third of that with Delta,^58,59^ with similar observations reported in France and Norway.^60,61^ In the United States, the percentage of hospitalization, intensive care unit (ICU) admission, receipt of invasive mechanical ventilation (IMV), and in-hospital death were lower during the Omicron pandemic than during the Delta pandemic, and the mean length of hospital stay was considerably shorter.^62,63^ Moreover, a high rate of asymptomatic carriage has been observed since the discovery of the Omicron variant,^44^ which may suggest milder symptoms of the variant.

Additionally, preliminary laboratory data confirmed the lower pathogenicity of the Omicron variant compared with the earlier variants. The formation of multinuclear syncytia is a significant pathological step in COVID-19 infection, reflective of cell-cell fusion events during viral infection.^64–66^ In vitro assays have shown that the Omicron variant poorly induced multinuclear syncytia in multiple cell lines^27,52^ compared with previous variants.^67^ Moreover, higher cell viability was observed in Omicron variant-infected cells compared with those infected with previous variants,^32^ which was consistent with findings in vivo. Infection with the Omicron variant caused limited bodyweight loss, lower viral load in the upper and lower respiratory tracts, and limited lung pathological damage and mortality rates compared with earlier variants in both hamsters or human ACE2 (hACE2) transgenic mice.^32,52,53^ In addition, the Omicron variant was less effective in antagonizing cellular interferon signaling compared with the Delta variant. Moreover, the activation of the NF-κB pathway is less efficient in response to the Omicron variant,^68^ implying that Omicron may induce slighter inflammatory responses.^34^

The evidence above further confirms that the Omicron variant is less severe than previous variants. However, it is possible that the pathogenicity of the Omicron variant may be underestimated because of rising levels of herd immunity via previous infections and vaccinations.^69–71^ Notably, the proportion of young patients was higher among those infected with Omicron,^57,72^ which may result in reduced pathogenicity. In addition, the impact of Omicron is not attenuated by reduced pathogenicity, the healthcare system is still under enormous pressure because of the high transmissibility of the Omicron variant.^54,73^

## Immune evasion

The most serious concern about the Omicron variant is its high immune evasion ability. The Omicron variant can escape the immune response established by vaccination or previous infection by other variants, which may result in high transmissibility.^11,16,71,74–79^ Data from the UK showed that BA.2 and BA.1 have similar immune evasion abilities.^41,80,81^ Moreover, a recent study showed that BA.2 can re-infect BA.1 convalescent patients, despite the small number of such cases identified.^80^

There are several mutations in the RBD region and N-terminal domain (NTD) of the Omicron variant, which are the main targets of neutralization.^82–85^ Unprecedented complexity in mutation patterns can alter antigenicity, invalidating the existing immunity.^86^ The cryo-electron microscopy (cryo-EM) structure helps to reveal the basis of immune evasion by Omicron. Omicron S-trimer exclusively formed one conformational state with one “up” RBD and two “down” RBDs, while a single-up conformation and all-down conformation were depicted in previous variants.^15,87^ Steric clashes, altered interactions at antibody-binding surfaces, and local changes in the spike structure were induced by mutations that interfered with antibody recognition.^86,87^

### Vaccines

**Fig. 3:**
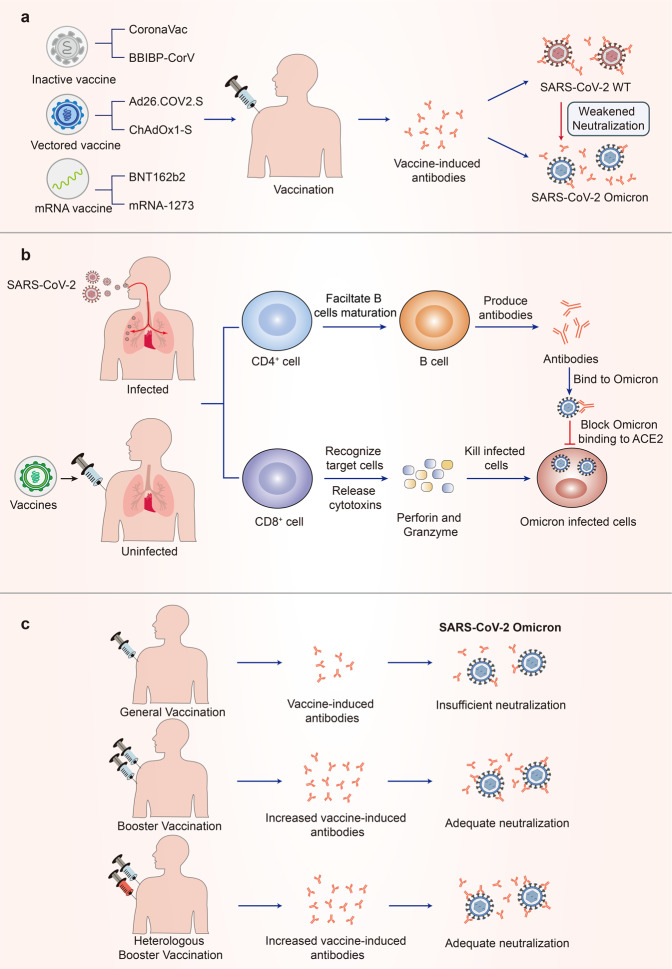
Vaccine-induced immunity against SARS-CoV-2 Omicron. **a** SARS-CoV-2 Omicron escapes vaccine immunity. The major vaccine candidates targeting SARS-CoV-2 induce antibodies after vaccination that can neutralize SARS-CoV-2 WT but are less effective against Omicron. **b** T-cell immune responses induced by SARS-CoV-2 infection or vaccination are effective against Omicron infection. **c** Booster vaccination or Heterologous booster vaccination can induce increased antibodies, which can provide adequate neutralization against Omicron

Since the outbreak of the COVID-19 pandemic, some vaccines have been developed to end the pandemic; however, these vaccines have been less effective against the Omicron variant. Available evidence shows that the major vaccines in use around the world are significantly less effective against Omicron. The neutralization activity against Omicron was below the lower limit of quantitation in over 80% of serum samples from individuals who received two doses of inactivated vaccines: CoronaVac and BBIBP-CorV.^88–92^ Moreover, Ad26.COV2.S, ChAdOx1-S, and Sputnik V, representative of vectored vaccines, failed to trigger effective neutralizing activity against the Omicron variant.^16,93–95^ RNA vaccines, such as BNT162b2 and mRNA-1273, which were proven to be the most effective against the WT strain, were completely ineffective against the Omicron variant in over 50% of individuals.^92,93,95–102^ In serum samples from individuals who had received two doses of BNT162b2 or mRNA-1273, there was a considerable decrease in the titers of neutralizing antibodies against the Omicron variant compared with the WT.^93,94,96–98,103–106^ Although each study differed due to differences in samples and testing methods, the findings showed that there was a considerable decrease in the neutralizing potency of two doses of RNA vaccines against Omicron. An observational study in the United States showed that the estimated effectiveness of two doses of RNA vaccines against Omicron was only ~30% 1 month after the second dose, with no effectiveness three months after the second dose for BNT162b2 and 6 months after the second dose for mRNA-1273.^107^ Data on other vaccines against Omicron variants have not been disclosed (Fig. 3a).

The cellular immune response is a major determinant of the clinical outcome of SARS-CoV-2.^108^ Multiple mutations in the spike protein of the Omicron variant contribute to escape from antibody neutralization, but components of the cellular immune response, such as T cells, can still target Omicron and provide protection from severe outcomes. Studies have shown that cellular immunity induced by vaccination or previous infection is highly conserved in the SARS-CoV-2 Omicron Spike.^109–114^ The median relative frequencies of SARS-CoV-2 spike-specific CD4^+^ T cells and CD8^+^ T cells that cross-recognize Omicron are ~70–90% in previously infected or vaccinated individuals,^110–113^ although there is still more than 50% reduction in 20% of individuals.^114^ In addition, booster vaccination enhances T-cell responses to Omicron spike.^109,114^ In vaccines, a median of 11 and 10 spike epitopes were recognized by CD4^+^ and CD8^+^ T cells in T-cell epitope repertoire analysis, respectively, with over 80% preservation for Omicron at the epitope level, which retained binding to HLA-I^111,114^ (Fig. 3b).

Although routine doses of vaccination are rarely effective in neutralizing the Omicron variant, substantial evidence has shown that booster vaccination can induce neutralizing immunity against Omicron more effectively.^16,77,115^ Post-booster vaccinations have shown a dozen-fold increase in neutralization titers for Omicron, indicating a significant reduction against the Omicron variant compared to the WT strain.^88,93,96,99–101,116^ Moreover, administering three doses of BNT162b2 and mRNA-1273 vaccines increased effectiveness against Omicron to ~65% and 72%, respectively, compared with unvaccinated mice.^107^ Interestingly, heterologous booster vaccination has the potential to induce adequate neutralizing efficacy,^89,92,117,118^ which is important for augmenting the protection provided by some vaccines with insufficient neutralizing antibodies. For instance, heterologous booster vaccination with aerosolized Ad5-nCoV, a representative of adenovirus-vectored vaccines, generated greater neutralizing antibody responses against the Omicron variant than that generated by homologous booster vaccination with CoronaVac,^119^ which provided an effective alternative in response to the Omicron. However, there is no evidence yet that heterologous vaccination is superior to homologous vaccination,^120^ and the protective efficacy obtained could be related to the type of vaccine. Therefore, further research on the mechanism and safety of heterologous vaccination is necessary, which could help solve the problem of vaccine shortage (Fig. 3c).

Overall, routine doses of vaccination can rarely provide adequate protection against the Omicron variant; therefore, homologous or heterologous booster vaccinations are necessary, which also highlights the significance of the supply and equitable distribution of vaccines. In addition, the plasma of convalescent individuals can hardly neutralize the Omicron variant, although cross-neutralization has been observed against earlier variants.^77,91,95,115^ However, infection plus vaccination can induce high-quality antibodies with superior neutralization capacity.^91,94,95,99,103,116,121–123^ Given a large number of infected individuals, this group of people may only require a routine vaccination dose to obtain effective protection against Omicron.

### Therapeutic-neutralizing antibodies

In this section, we summarized the effect of neutralizing antibodies from vaccines and previous infections against the Omicron variant. The neutralizing ability of therapeutic-neutralizing antibodies against Omicron is weak. Most therapeutic-neutralizing antibodies with EUA approval or at advanced clinical development stages have failed to neutralize the Omicron variant (Table 1).^16,75–77,106,115,124–126^ Neutralizing antibodies can be divided into two groups. The first group contains the vast majority of neutralizing antibodies, including LY-CoV555 (marketed as bamlanivimab), LY-CoV016 (marketed as etesevimab), REGN10987 (marketed as imdevimab), REGN10933 (marketed as casirivimab), COV2-2196 (marketed as tixagevimab), COV2-2130 (marketed as cilgavimab) and CT-P59 (marketed as regdanvimab), which block binding of Spike protein to the receptor ACE2.^16^ Most of these antibodies lost their neutralizing ability against Omicron due to the destruction of the antigenic epitope.^16,75,76,124,126^ K417N, E484A, Q493R, and N501Y are the main sites responsible for the evasion.^15^ The combination of COV2-2196 and COV2-2130 exhibits neutralizing activity against Omicron; however, its neutralization ability against Omicron is 12–200-fold lower compared with that against the WT,^16,115,124–127^ due to the N440K and G446S mutations.^76,115,127^ However, this combination, especially COV2-2130, has been reported to retain activity against BA.2, perhaps due to the absence of G446S.^128^ The second group, represented by VIR-7831/S309 (marketed as sotrovimab), rarely competes with ACE2 but recognizes non-RBM epitopes that are conserved within sarbecoviruses, including SARS-CoV.^129^ S309 shows resistance to Omicron’s antibody escape, with only a two- to threefold decrease in neutralization efficiency for Omicron compared with the WT.^16,76,115,124,126,127^ G339D and N440K are presumed to interfere with the combination of S309 and spike protein due to the proximity of the antigenic site, but the impact is limited.^76,127^ BA.2 seems to have a greater negative impact on S309 because of the S371F mutation.^128^ Despite the presence of the S371L mutation in BA.1, S371F in BA.2 displayed distinct resistance to neutralizing antibodies compared to S371L.^128^ Brii-198 (marketed as romlusevimab) possesses neutralization efficiency against Omicron, but the neutralization ability is slightly weaker and can be inhibited by R346K.^75^ In addition, S2X259 possesses considerable neutralizing potency against the Omicron variant,^16^ but was less effective against the BA.2 strain.^128^ LY-CoV1404 (marketed as bebtelovimab), which has recently received EUA approval, possesses a high neutralization capacity against all identified variants.^128,130^ Structural analysis demonstrated that the epitope of LY-CoV1404 was highly conserved, except for N439 and N501. Fortunately, the N501Y mutation of Omicron does not interfere with its binding capacity.^130^

**Table 1:** Neutralizing antibodies against SARS-CoV-2 Omicron variant

Stages	Antibody name	Company	Neutralizing effect compared to SARS-CoV-2 WT	Mutations affecting neutralizing efficiency	Refs.
EUA (combined with etesevimab)	LY-CoV555 (bamlanivimab)	Lilly	Completely lost its activity	E484A, Q493R	^75^
EUA (combined with bamlanivimab)	LY-CoV016 (etesevimab)	Lilly	Completely lost its activity	S371L, K417N, Q493R; S371F, D405N	^75,128^
EUA	LY-CoV1404 (bebtelovimab)	Lilly	Almost identical	—	^128,130^
EUA (combined with casirivimab)	REGN10987 (imdevimab)	Regeneron Pharmaceuticals	Completely lost its activity	S371L, N440K, G446S; S371F	^75,115,128^
EUA (combined with imdevimab)	REGN10933 (casirivimab)	Regeneron Pharmaceuticals	Completely lost its activity	S371L, K417N, S477N, E484A, Q493R; S371F	^75,115,128^
EUA (combined with cilgavimab)	COV2-2196 (tixagevimab)	AstraZeneca	hundreds of times reduced	E484A, Q493R; S371F	^16,128^
EUA (combined with tixagevimab)	COV2-2130 (cilgavimab)	AstraZeneca	hundreds of times reduced in BA.1, but remained largely active in BA.2	R346K; G446S	^16,128^
EUA	VIR-7831/S309 (sotrovimab)	GSK & Vir Biotechnology	2–3-fold reduced in BA.1, but dozens of times reduced in BA.2	G339D, S371L; S371F	^75,127,128^
Stage III	CT-P59 (regdanvimab)	Celltrion	Completely lost its activity	K417N, G446S, E484A, Q493R	^16,127^
Stage III	Brii-196 (amubarvimab)	Brii Biosciences	hundreds of times reduced	S371L, Q493R; S371F	^75,128^
Stage III	Brii-198 (romlusevimab)	Brii Biosciences	Maintains activity in BA.1, but is inhibited by R346K	R346K; S371F	^75,128^
Stage III	ADG20 (adintrevimab)	Adagio Therapeutics, Inc.	20–200-folds reduced in BA.1, but almost lost activity in BA.2	S371L; S371F	^75,77,115,128^
Stage I	DXP-604	SINGLOMICS	About thirty times reduced	—	^76^
Preclinical	S2X259	Humabs Biomed SA-Swiss Biotech	About ten times reduced in BA.1, and hundred times reduced in BA.2	S371L; S371F	^75,128^
Preclinical	S2K146	Vir Biotechnology	Basically retained activity	K417N, T478K, E484A, Q496S	^16^
Preclinical	S2H97	Vir Biotechnology	Basically retained activity	—	^16^

Overall, neutralizing antibodies targeting the conserved epitopes of SARS-CoV-2 variants and other sarbecoviruses may have broad prospects for the control of COVID-19 pandemic. Given that SARS-CoV-2 is prone to mutations, developing neutralizing antibodies targeting relatively conserved sites is an effective option to deal with emerging variants, such as Omicron. There are still some doubts about the efficacy of the antibodies mentioned above in treatment. A randomized controlled trial indicated that neither S309 nor Brii-198 showed efficacy in improving clinical outcomes despite the small sample size.^68^ Moreover, the efficacy of LY-CoV1404 is yet to be validated in clinical studies.

## Antiviral drugs and potential treatments

**Fig. 4:**
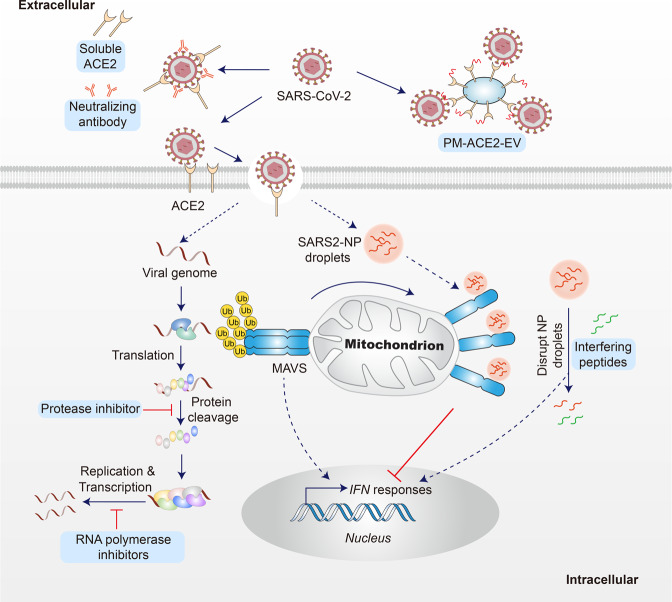
Mechanisms of antiviral drugs and potential treatments against SARS-CoV-2 Omicron. Extracellularly, soluble ACE2, neutralizing antibodies, and palmitoylated ACE2-enriched EVs can capture SARS-CoV-2 viruses and inhibit SARS-CoV-2 interaction with cell-surface ACE2, resulting in reduced infection. Intracellularly, on the one hand, the released viral genome is translated to produce the polyproteins, which are cleaved by proteases to yield the RNA replicase–transcriptase complex. Then viral genome is duplicated and mRNA encoding structural proteins are transcribed. The protease inhibitors and RNA polymerase inhibitors can be used to inhibit the process of cleavage, transcription, and replication. On the other hand, the N protein of SARS-CoV-2 undergoes LLPS with RNA, which inhibits the aggregation and Lys-63-linked poly-ubiquitination of MAVS and thereby suppresses the innate antiviral immune response. The usage of interfering peptides disrupting N protein droplets can restore the impaired immune response

In addition to vaccines and therapeutic-neutralizing antibodies, several antiviral drugs and potential treatments are being investigated for use against Omicron. Antibody therapies have been shown to be less effective against the Omicron variant; however, the situation seems different for antiviral drugs. In vitro assays have confirmed that Omicron is susceptible to most antiviral drugs under investigation, including remdesivir, molnupiravir, PF-07304814 (nirmatrelvir, a key component of paxlovid), EIDD-1931, ribavirin, favipravir, nafamostat, camostat, and aprotinin.^68,126,131^ These drugs have different mechanisms of action, indicating that the drug sensitivity profile of the Omicron variant does not change considerably in response to the drugs. There are only two missense mutations in the replicase–transcriptase complex (nsp7-10, nsp12, nsp14) of the Omicron variant, which may have little effect on RNA-dependent RNA polymerase-inhibitor drugs, such as remdesivir and molnupiravir.^68^ However, current research is still at the cellular level, and more clinical results are needed to support these conclusions. Moreover, the efficacy of several drugs is yet to be proven against Omicron. There is an urgent need to develop new broad-spectrum antiviral drugs in response to changing viruses and the current shortage of vaccines and drugs. Drugs that inhibit viral replication are likely to remain effective against the Omicron variant, which is also the mechanism of drugs that inhibit the binding of viruses to ACE2. The ACE2-targeting antibody has been shown to suppress the Omicron variant at the cellular level,^132^ which is a likely direction of exploration. Boosting the antibody responses using biochemical methods is also a method that could be explored. A recent study showed that an ultrapotent RBD-targeted biparatopic nanobody exhibited enhanced neutralizing activity against Omicron.^133^ In addition, it has been reported that engineered extracellular vesicles (EVs) enriched with palmitoylated ACE2 (PM‐ACE2) efficiently captured SARS‐CoV‐2 viruses and inhibited their interaction with cell surface ACE2, leading to reduced infection both in vitro and in vivo.^134^ Furthermore, it has been demonstrated that patients with COVID-19 exhibit impaired IFN responses, especially in patients with severe disease, which might contribute to the limited antiviral response.^135–138^ Therefore, targeting antiviral responses could be important in managing SARS-CoV-2. A recent study showed that the N protein of SARS-COV-2 inhibits the activity of MAVS, a key component of antiviral innate immunity, by undergoing liquid-liquid phase separation (LLPS) with RNA, thereby suppressing the antiviral immune response.^139^ Interestingly, the 246–365 domain of the N protein, which retains the phase-separation ability, is hardly mutated in the currently reported SARS-CoV-2 variants, including Omicron. Thus, targeting the SARS2-N protein LLPS could be a promising therapeutic approach against infection WT and variants of SARS-CoV-2. These potential treatments may help curb ongoing pandemics as well as any future outbreaks (Fig. 4).

## Conclusions and perspectives

The Omicron variant has attracted worldwide attention since its emergence owing to the high number of mutations, which increased its transmissibility and immune evasion capability. Although the binding ability of Omicron to ACE2 is still controversial, it has enhanced transmissibility, making it the dominant species in several regions within a short period of time. Omicron spikes inefficiently utilize TMPRSS2 to enter cells, but mainly rely on the endocytic pathway, which leads to a decrease in replication in the lung parenchyma and an enhanced ability to infect the upper respiratory tract,^27,31,32^ making the virus less pathogenic. Routine vaccination or a previous infection cannot provide effective protection against Omicron, and booster vaccination is required. Additionally, only a few neutralizing antibodies are active against Omicron, whereas most antiviral drugs in development are effective against it.

In the wake of the Omicron variant, it was believed that the effect would be minimal due to herd immunity established by vaccination and infection, and the development of specific drugs. Moreover, the discovery of the Omicron variant indicates that the attempt to end the COVID-19 pandemic might be hindered by the tendency of the virus to mutate. The major research focus should be the development of vaccines and drugs against possible variants of the virus.

Since the outbreak of the Omicron variant, several studies have been performed to improve our understanding of the mechanism and characteristics of the variant. Currently, vaccines, social distancing, and specific drugs are still effective means of resisting Omicron. Homologous or heterologous boosters and new vaccines against Omicron, and possibly new variants, have been proposed to improve protection in response to vaccine failure. However, early animal experiments have shown that Omicron-targeted vaccines were not more effective than booster jabs of already developed vaccines against the variant, although they all generated broad antibody responses to all variants, including Omicron.^140–142^ In naive mice immunized with Omicron-matched vaccines, high levels of potent antibodies against Omicron were produced, but their ability to inhibit previous variants was limited.^142,143^

Although it is believed that the omicron variant may likely not be the last mutant, it is expected that its effect will decrease with increasing immunity among the populace due to vaccines and infections. A previous study indicated that Omicron infection of vaccinated individuals, but not unvaccinated individuals, increases neutralizing activity against the Delta variant,^144^ indicating that the occurrence of previous variants is likely to reduce. Although no novel mutant has been discovered, the general trend is that the virus is becoming less severe, mainly because of enhanced immunity.

Furthermore, to effectively combat the Omicron variant and the pandemic in general, it is important to emphasize equal distribution of vaccines, especially in underdeveloped and developing regions. Presently, the three-injection vaccine can provide effective protection against Omicron, although there will be a percentage of breakthrough infections with milder symptoms. Some countries have approved the implementation of the fourth-injection vaccines. Recent researches show that the protection provided by the third dose of vaccines wanes over time, while the antibody levels can be restored by a fourth dose.^145^ Therefore, it is beneficial to administer a fourth dose of the vaccine to certain groups such as the elderly and immunocompromised individuals.^146,147^ Nevertheless, it is more crucial to complete the three doses of vaccination in a larger population than the fourth dose in healthy individuals, although we may need more boosters to maintain antibody levels in the long run. Furthermore, social distancing restrictions should not be lifted prematurely, as this can lead to unpredictable consequences.
